# Negative capacitors and inductors enabling wideband waveguide metatronics

**DOI:** 10.1038/s41467-023-42808-z

**Published:** 2023-11-03

**Authors:** Xu Qin, Pengyu Fu, Wendi Yan, Shuyu Wang, Qihao Lv, Yue Li

**Affiliations:** 1 Department of Electronic Engineering, Tsinghua University, Beijing, 100084 China; 2 Beijing National Research Center for Information Science and Technology, Beijing, 100084 China

**Keywords:** Electrical and electronic engineering, Electronic devices, Metamaterials

## Abstract

Waveguide metatronics, known as an advanced platform of metamaterial-inspired circuits, provides a promising paradigm for millimeter-wave and terahertz integrated circuits in future fifth/sixth generation (5/6G) communication systems. By exploiting the structural dispersion properties of waveguides, a lumped type of waveguide integrated elements and circuits could be developed in deep subwavelength scales with intrinsic low loss and low crosstalk. In this study, we focus on constructing negative capacitors and inductors for waveguide metatronics, effectively expanding the operating frequency range of waveguide integrated circuits. The incorporation of negative elements enables wideband impedance matching in waveguide, which have been both theoretically explored and experimentally validated within the waveguide metatronics paradigm. Furthermore, we have demonstrated that the negative elements can also be realized in the optical domain through the utilization of a silicon waveguide with photonic crystal cladding, indicating the feasibility and universality of wideband waveguide metatronics. The negative lumped elements could boost the progress of the waveguide metatronic technique, achieving superior performance on the conventional lumped circuits within waveguides that solely rely on positive elements.

## Abstract (web summary)

The authors showcase a method to design negative lumped elements by engineering the effective permittivity within the waveguide, which enables realizations of wideband waveguide metatronics and promises performance enhancement in various fields.

## Introduction

In the field of electronics, modularization and integration of individual functional circuits have effectively simplified the existing electronic systems and played a pivotal role in the development of integrated circuits^1^. Inspired by electronic circuits, the concept of metatronics has been introduced to explore a metamaterial-inspired method for light manipulating and tailoring with lumped elements in subwavelength scales^2,3^. This innovative mechanism has boosted various optical lumped circuits including optical filters^4–7^, nanoantennas^8–11^, transmission structures^12–14^, metasurfaces^15^, Wheatstone bridges^16^, and photoelectric computation^17,18^. This concept of metatronics presents a distinctive and feasible paradigm for optical integrated circuits, paving the way for the realization of nanoscale devices for light.

In recent years, an advanced form of metatronics is proposed by taking advantage of the dispersion of the waveguides and is known as waveguide metatronics^19^. Waveguides provide a practical platform for optical metatronics by performing waveguide integrated circuits with lumped elements, emancipating the implementation of the optical metatronics from the limitations of adopting intrinsic plasmonic materials at optical frequencies. Moreover, at terahertz frequencies, the metallic waveguides possess the merits of low crosstalk between multipath signals and significantly lower transmission loss compared to coplanar transmission lines or low-profile microstrip lines^20,21^. In the paradigm of waveguide metatronics, various practical applications and devices have been proposed, including filters^22,23^, impedance matching^24,25^, active circuits^26^, etc. The increasingly sophisticated techniques in the waveguide platform have promised impressive potential in future microwave and terahertz systems such as 5/6G communications^27,28^.

**Fig. 1:**
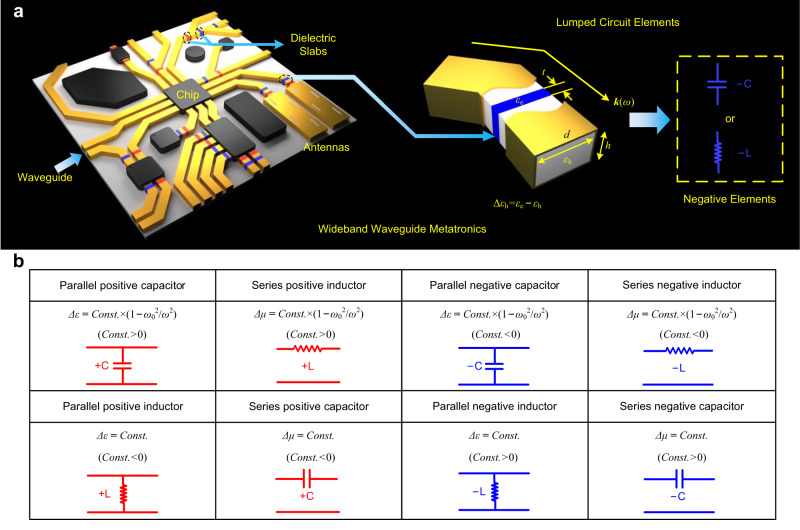
Concept of wideband waveguide metatronics with proposed negative elements. **a** Concept and element map including positive and negative lumped elements for wideband waveguide metatronics (${\omega }_{0}$ is the cutoff frequency of the waveguide). **b** A comprehensive map of the lumped elements for waveguide metatronics.

Here, we have introduced the exotic negative lumped elements inside waveguides to expand the operation frequency range of the waveguide metatronic circuits. Figure 1a illustrates the general concept of a waveguide metatronic circuit. Within the waveguide conduit, we strategically design the dielectric slabs (depicted in red and blue) with different permittivity from the host medium, creating a discontinuity along the waveguide. The electromagnetic response of the discontinuity could be modularized as a lumped element, such as an inductor or a capacitor, with red indicating positive elements and blue representing negative elements. Due to the waveguide structural dispersion, the effective permittivity with a properly designed value is acquired according to the theory of waveguide effective plasmonics^29,30^. Therefore, in this work, we propose a method to engineer a lumped element with negative inductance or capacitance by engineering the effective permittivity. Based on the negative lumped elements, we have realized the wideband waveguide metatronics distinguished from the regular ones in ref. ^19^. Moreover, the proposed negative elements are based on the structural dispersion of the waveguide rather than on the plasmonic material dispersion only^31^, thus promising a wide range of unprecedented wideband applications in the waveguide integrated circuits. To demonstrate the potential of the negative lumped elements, wideband impedance matching inside waveguides has been theoretically explored and experimentally verified, exhibiting superior performance to the conventional waveguide metatronic circuits comprising only positive elements. Moreover, we validate the feasibility of these negative lumped elements and circuits at optical frequencies using a silicon waveguide with photonic crystal cladding, indicating the universality of this technique. The proposed wideband waveguide metatronics incorporating negative lumped inductors and capacitors exhibit exciting feasibility for future millimeter-wave and terahertz integrated circuits and systems.

## Results

Figure 1b exhibits a comprehensive map of the lumped elements for waveguide metatronics, including the positive/negative capacitor/inductor with series/parallel configuration, which could be properly designed in the paradigm of waveguide metatronics. The response of the discontinuity caused by the inserted dielectric slab in the waveguide could be modularized as a lumped element with the normalized admittance value as follows:

1 $${{{{{\rm{y}}}}}}=1+\frac{j\omega \varDelta \varepsilon {\varepsilon }_{0}t}{\sqrt{{\varepsilon }_{{{{{{\rm{eff}}}}}}}}}\sqrt{\frac{{\mu }_{0}}{{\varepsilon }_{0}}}=1+\frac{j\omega \varDelta \varepsilon {\varepsilon }_{0}t}{\sqrt{{\varepsilon }_{{{{{{\rm{h}}}}}}}-\frac{{\pi }^{2}{c}^{2}}{{d}^{2}{\omega }^{2}}}}\sqrt{\frac{{\mu }_{0}}{{\varepsilon }_{0}}}$$

in which ${\varepsilon }_{{{{{{\rm{eff}}}}}}}={\varepsilon }_{{{{{{\rm{h}}}}}}}-\frac{{\pi }^{2}{c}^{2}}{{d}^{2}{\omega }^{2}}$ represents the effective permittivity of the host medium in the waveguide according to waveguide effective plasmonics^29,30^. $\varDelta \varepsilon={\varepsilon }_{{{{{{\rm{e}}}}}}}-{\varepsilon }_{{{{{{\rm{h}}}}}}}$ is the permittivity difference in the discontinuity, ${\varepsilon }_{{{{{{\rm{e}}}}}}}$ and ${\varepsilon }_{{{{{{\rm{h}}}}}}}$ are the relative permittivities of the inserted dielectric slab and the host medium, respectively, $t$ is the thickness of the slab, $d$ is the width of the waveguide, $c$ is the speed of light in a vacuum, $\omega$ is the operating frequency, ${\varepsilon }_{0}$ and ${\mu }_{0}$ are the permittivity and permeability in vacuum, respectively. The inserted dielectric slab with varying permittivity lead to different responses inside the waveguide, and as shown in Fig. 1b, a positive or negative inductor is achieved with $\varDelta \varepsilon=Const.$, while when $\varDelta \varepsilon=Const.\times (1-{\omega }_{0}^{2}/{\omega }^{2})$ we could obtain a positive or negative capacitor, where ${\omega }_{0}$ is the cutoff angular frequency. On the other hand, according to the dual theorem, lumped series elements, serving as the counterpart of lumped parallel elements, are realized by the inserted dielectric slabs with different permeability from the host medium inside the waveguide. The detailed theoretical derivations and analyses are demonstrated in Supplementary Note 1. The negative circuit elements possess unique properties, and manifest remarkable wideband performance which has never been realized with only positive elements. Here, we aim to utilize negative elements to realize wideband impedance matching inside the waveguide, consequently achieving better performance than conventional waveguide metatronic circuits using only positive elements.

**Fig. 2:**
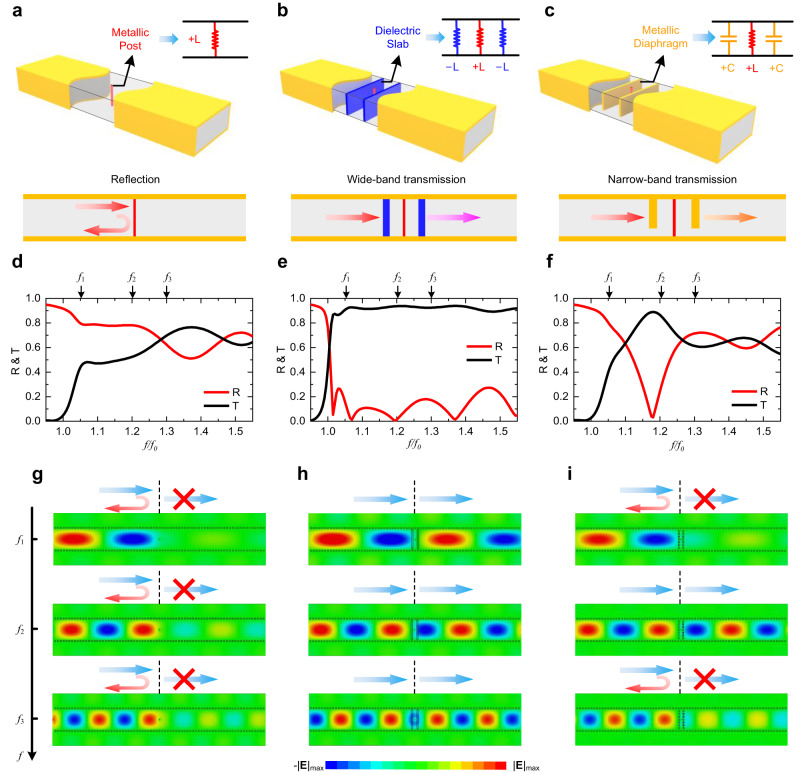
The performance of the lumped elements for wideband impedance matching. **a** Inductive metallic post in waveguide. **b**, **c** Electromagnetic impedance matching for metallic post with positive and negative elements, respectively. **d**–**f** Performance of the metallic post in waveguide and electromagnetic impedance matching by different elements. **g**–**i** Numerical field distributions at different frequencies of the metallic post in waveguide and impedance matching by different elements.

As shown in Fig. 2a, a metallic post connecting the top and the bottom of the waveguide is positioned in the waveguide, which is known to represent an inductive load in a waveguide^20^. The presence of an inductive post would lead to obvious scattering and reflection in waveguide transmission. To eliminate the scattering effects, it is intuitive to introduce a capacitive load to match the impedance. At the capacitor-inductor resonant frequency, the impedance of the metallic post can be matched. Here, the negative inductors are involved as an alternative choice to compensate for the discontinuous impedance of this inductive post in a wide frequency range. In Fig. 2b, c, the metallic post is matched by the negative inductor and the positive capacitor, respectively. In Fig. 2b, two dielectric slabs are inserted on both sides of the metallic post, with the permittivity of the dielectric slabs properly chosen according to the elements map to realize negative inductors. In Fig. 2c, two metallic diaphragms are positioned on both sides of the metallic post and are only connected to the top of the waveguide, acting as positive capacitive loads^20^. Figure 2d–f depict the reflection and transmission coefficients (R & T) with the cutoff frequency *f*_0_ = 2 GHz, illustrating the impact of the metallic post and the impedance matching performance achieved by the negative inductor and the positive capacitor. As shown in Fig. 2d, the metallic post leads to significant reflection in the waveguide, with the reflection coefficient exceeding 0.5 across the observed frequency range. In Fig. 2e, the negative inductors realize a wideband impedance matching with the positive inductive post and achieve the total transmission over a wide frequency range. Due to the loss of the host medium ($\tan \delta=0.002$) and the imperfect metal conductivity of copper, the transmission coefficient does not reach unit. In a frequency range with a relative bandwidth of over 43.14% (relative bandwidth refers to the ratio of the absolute bandwidth to the center frequency in the operating band), the transmission coefficient is increased to higher than −3 dB (i.e., T > 0.707, indicating that more than half of the energy is transmitted). While in Fig. 2f, the positive capacitors also realize impedance matching of the post at the resonant frequency of the inductor and capacitor. However, the relative bandwidth with a transmission coefficient higher than −3 dB is limited to 10.57%, which is far narrower than the bandwidth achieved with negative elements, and it is exactly what we have expected. To provide an intuitive understanding of the wideband performance enabled by negative elements, Fig. 2g–i exhibit the electric field distributions at three different frequencies. The impedance matching achieved with negative inductors realizes total transmission at all observed three frequencies. While the results obtained with positive capacitors perform well only around *f*_2_, which corresponds to the capacitor-inductor resonant frequency. The involvement of the negative elements has improved the relative transmission bandwidth from 10.57% to 43.14%.

**Fig. 3:**
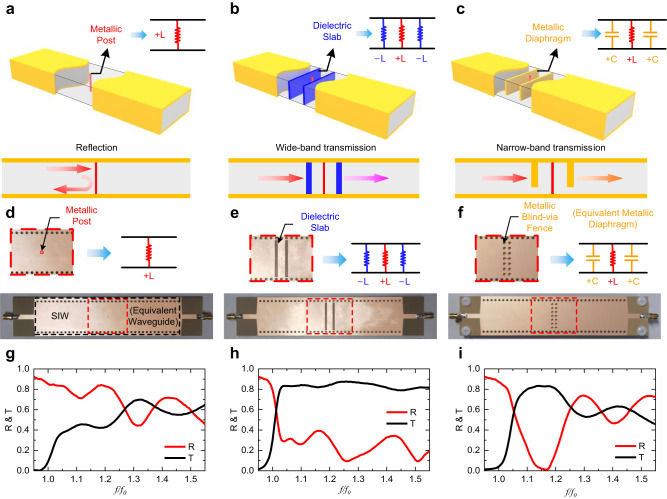
Experimental results of the impedance matching in waveguide metatronics. **a**–**c** Inductive metallic post in waveguide with positive and negative elements. **d**–**f** Experiment prototypes for the post in waveguide with positive and negative elements. **g**–**i** Experimental results of the reflection and transmission in the corresponding prototypes.

To validate the concept of wideband waveguide metatronics and verify the unique effect of negative elements, the corresponding circuit prototypes are fabricated and experimentally tested. Figure 3a–c shows three circuits including the scattering of a single positive inductor, electromagnetic impedance matching with the negative inductor, and electromagnetic impedance matching with the positive capacitor, respectively. As exhibited in Fig. 3d–f, the prototype fabrication is conducted using standard printed circuit board (PCB) technology. In Fig. 3d, the substrate-integrated waveguide (SIW)^32^ is adopted to construct an equivalent waveguide on a single layer of PCB. A periodic metallic via-hole fence is employed as a metallic wall connecting the top and the bottom of the waveguide^33^, behaving as the lateral metallic boundary of the waveguide to realize SIW. Moreover, metallic blind-hole fences only connecting to the top of the waveguide were utilized in Fig. 3f to emulate equivalent metallic diaphragms. Details of the prototypes and experimental setup are described in Supplementary Note 2. Figure 3d–f display the experimental performances of three groups of waveguide metatronic circuits. As previously concluding, the single positive inductor in the waveguide lead to obvious reflection and signal transmission deterioration. The positive capacitive elements realize impedance matching of the inductive post but only in a limited −3 dB relative bandwidth of 13.62%. In comparison, the results obtained with negative inductors demonstrate a broad relative bandwidth of over 41.36%. The experimental results succeed in verifying the unique properties and the exciting wideband performance of the negative elements in waveguide metatronics, distinguishing them from the conventional positive ones.

### Impedance properties of lumped negative elements in waveguide metatronics

**Fig. 4:**
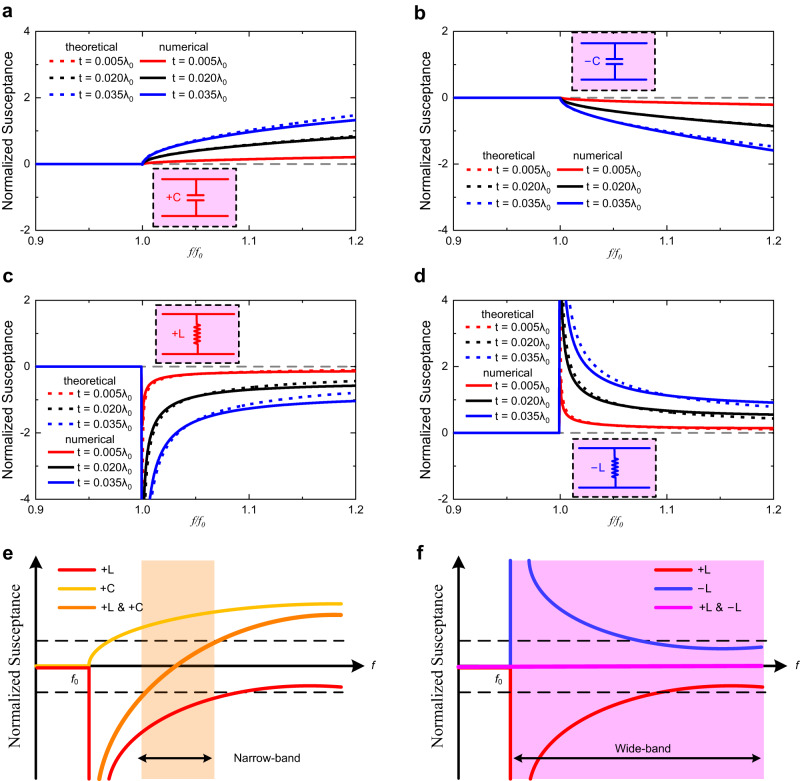
Discussion of the elements performance and comparison between positive and negative elements. **a**–**d** Normalized susceptance of the positive capacitor, negative capacitor, positive inductor and negative inductor, respectively. **e**, **f** Sketches and analysis of the bandwidth performance with positive and negative elements.

To further clarify the bandwidth enhancement achieved with the proposed negative elements, their intrinsic impedance properties have been investigated and demonstrated. The impedance of the lumped element in waveguide metatronics is primarily determined by the thickness of the dielectric slab $t$ and the permittivity difference between the slab and host medium $\varDelta \varepsilon$^19^. Different dispersions of $\varDelta \varepsilon$ could construct different types of elements, with the impedance proportional to the thickness $t$. Here, we have exhibited the positive/negative capacitor/inductor with different thicknesses of 0.005${\lambda }_{0}$, 0.020${\lambda }_{0}$, 0.035${\lambda }_{0}$, where ${\lambda }_{0}$ represents the free-space wavelength. Figure 4a–d show results of the cases where $\varDelta {\varepsilon }_{+{{{{{\rm{C}}}}}}}=+ 25(1-{\omega }_{0}^{2}/{\omega }^{2})$, $\varDelta {\varepsilon }_{-{{{{{\rm{C}}}}}}}=-25(1-{\omega }_{0}^{2}/{\omega }^{2})$, $\varDelta {\varepsilon }_{+{{{{{\rm{L}}}}}}}=-5.15$, $\varDelta {\varepsilon }_{-{{{{{\rm{L}}}}}}}=+ 5.15$. Here, the material dispersion is determined from the theoretical analysis to construct desired negative elements. Although some of the desired material dispersions have hardly been discussed due to the lack of immediate applications, we aim to provide the targets for the researchers on both electromagnetism and material science to notice and find such materials or physical structures with desired material dispersion. The numerical results in Fig. 4a–d show the consistency with the theoretical results. The positive and negative elements exhibit opposite normalized susceptance, and the numerical difference between the absolute values of positive and negative elements is less than 16.6% in the observed frequency ranges.

Based on the opposite admittance values of the positive and negative elements, there would be an intuitive prospect of the wideband impedance matching realization, as well as the nature of the performance gap between the proposed negative elements and the conventional positive elements. As shown in Fig. 4e, f, the positive capacitor could realize the impedance matching in a limited frequency range, for the dispersive properties of positive capacitor and positive inductor are not complementary, making the impedance matching only applicable around the capacitor-inductor resonant frequency. In contrast, the negative inductor, with opposite dispersion to the positive inductor, could exactly compensate for the impedance of the positive inductor. As a result, the electromagnetic impedance matching is achieved over a rather broad frequency range. In practical applications, as the frequency gradually increasing, the discrepancy between theoretical and experimental results also gradually increases, therefore limiting the impedance matching performance at a higher frequency, which has been also shown in Fig. 2e. Even though, the proposed negative elements have succeeded in achieving wideband impedance matching over 40% relative bandwidth.

### Wideband waveguide metatronics in optical regimes

**Fig. 5:**
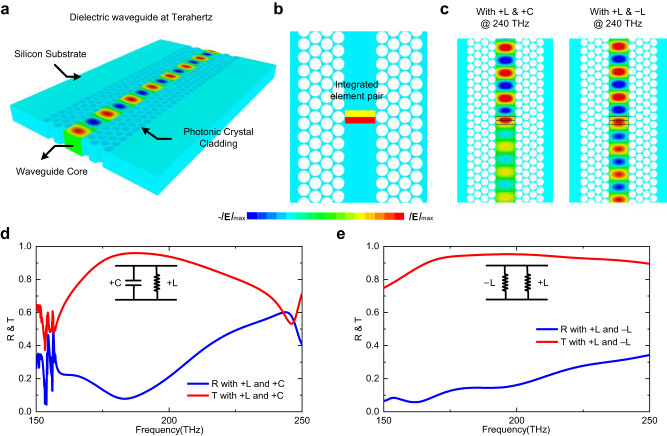
Wideband waveguide metatronics and negative elements in silicon-based waveguide at terahertz. **a** Structure of the optical waveguide in silicon substrate with photonic crystal cladding. **b** Waveguide metatronics in the silicon-based waveguide. **c** Field distributions in the waveguide with different circuit design. **d** Performance of the pair of positive capacitor and positive inductor in the silicon waveguide. **e** Performance of the compensated positive and negative elements in the silicon waveguide.

In the discussion above, the concept of negative circuit elements and wideband waveguide metatronics are independent of frequency scope. To exhibit the universality of the wideband waveguide metatronics, the feasibility of the concept in optical regimes has been explored. As shown in Fig. 5a, a dielectric waveguide is constructed in a silicon substrate with the photonic crystal cladding as waveguide boundaries^34–37^. Electromagnetic waves propagating in the core of the dielectric waveguide behave similarly to the TE_10_ mode, or behave as the quasi-TE_10_ mode, and therefore analysis in former sections are still applicable in the silicon dielectric waveguide. As shown in Fig. 5b, two dielectric slabs with different permittivity from the host silicon are inserted to build a circuit element pair at around 200 THz. Based on the circuit element pair, narrowband transmission could be achieved with a positive capacitor and a positive inductor, while wideband transmission could be achieved using a positive inductor and a negative inductor. The detailed element dimensions are demonstrated in Supplementary Note 3. As expected, the pair of positive capacitor and positive inductor only supports a narrowband transmission, while the pair of positive inductor and negative inductor achieves a total transmission in a rather wide frequency range. Figure 5c exhibits the electric field distributions of the two circuits in the waveguide core at a frequency of 240 THz. At this operating frequency, the pair of positive capacitor and positive inductor cannot support transmission, whereas the pair of positive inductor and negative inductor facilitates signal transmission with relatively low reflection. Figure 5d, e show their corresponding reflection and transmission coefficients, which are both consistent with our expectations. The relative bandwidth with a reflection coefficient lower than −10 dB (i.e., R < 0.316, indicating less than 10% energy reflection) for the wideband transmission with the pair of positive inductor and negative inductor is 46.47%. In comparison, the −10 dB relative bandwidth for the narrowband transmission with a positive capacitor and a positive inductor is 27.76%. These results have not only verified the wideband property achieved by negative elements, but also exhibited the feasibility and universality of wideband waveguide metatronics and negative elements in optical regimes, promising a broad prospect of the wideband waveguide metatronics in different types of waveguides and various operating frequencies.

As we have exhibited in Figs. 2 and 3, the functions of the proposed structure have been modularized to a lumped circuit element, and have further constructed lumped circuits in the waveguide. This analogy of lumped circuits has significantly simplified the functions of the proposed structure and built a bridge to the existing circuit paradigm. Here, we have theoretically and experimentally realized the negative elements integrated in the waveguide, and the feasibility as well as the performance of the lumped circuit elements have been verified in both metallic waveguide and optical silicon-based waveguide, which promises much potential in constructing integrated circuits in various waveguide systems. Furthermore, the proposed negative circuit element succeeds in realizing impedance matching in a rather wide frequency range, promising a large number of exciting performances in various fields, such as ultra-wideband cloaking. Generally speaking, cloaking exhibits a similar concept with impedance matching. In the circuit system, the reflection and transmission coefficients are described as scattering parameters, in which the reflection coefficient represents the total scattering waves in the waveguide. In the waveguide, the scattering waves are successfully removed in an unprecedented wide frequency band, which is indeed also a wideband cloaking in the waveguide. To some extent, the electromagnetic cloaking aims to a similar target with the wave impedance matching, i.e., removing the scattering waves introduced by possible obstacles. Therefore, the proposed concept of negative elements could bring more potential success in relative fields, especially for the promising applications of electromagnetic cloaking.

## Discussion

In this work, we have investigated the negative circuit elements in waveguide metatronics and further enhanced the operating frequency range of the waveguide metatronics. We have theoretically clarified and experimentally realized the negative circuit elements in waveguide metatronics, verifying their unique property to realize wideband impedance matching in the waveguide. Taking advantage of the negative lumped elements, impedance matching is achieved in a superior wide frequency range with a relative bandwidth over 40%. This represents a substantial improvement over the existing results with only positive elements. Moreover, the wideband waveguide metatronics with negative elements are also verified in silicon waveguides in optical regimes. We have verified the technique at around 200 THz and realized a wideband transmission with a bandwidth of 46.47%, indicating the feasibility and universality of the proposed concept in various frequency ranges and operating regimes. In conclusion, the proposed wideband waveguide metatronics hold great potential for future millimeter-wave and terahertz circuit systems such as 5/6G generation communications, and the intriguing negative circuit elements would promise superior performance in this advanced type of the waveguide metatronics.

## Methods

### Numerical full-wave simulations

The numerically simulated results in Figs. 2 and 4 were performed by full-wave simulations using the commercial software ANSYS HFSS® 18. In the simulation setup of Fig. 2, the host substrate is with a permittivity of 6.15 and a loss tangent angle of 0.0019. SIW is adopted to replace the metallic waveguide. The SIW is excited through two 50-Ohm microstrip lines at the two ends of the SIW. The 50-Ohm lumped ports are adopted at the two ends of the microstrip line, which are the position to connect the sub-miniaturized A (SMA) connectors, to excite the waveguide and connect the network analyzer. Copper is used in the model to mimic the perfect electric conductor (PEC) boundary with a conductivity of $5.8\times {10}^{7}\,{{{{{\rm{S}}}}}}\cdot {{{{{{\rm{m}}}}}}}^{-1}$. In the simulation setup of Fig. 4, the waveguide is the normal rectangular waveguide with the perfect electric conductor as boundary conditions. The host medium is with a permittivity of 6.15 and a loss tangent angle of 0. The waveguide is excited by two waveguide ports at two ends of the waveguide, and the normalized element value is derived from the S-parameters of the two waveguide ports. The numerically simulated results in Fig. 5 were performed by full-wave simulations using the commercial software CST STUDIO SUITE®. In the simulation setup of Fig. 5, a frequency-domain solver with tetrahedral meshing was adopted. The cells per max model box edge were set to 10. The waveguide is excited by waveguide ports, and the boundary of the simulated region is set to be open boundaries in all directions except the excitations to mimic an open system in free space. The host medium of the silicon waveguide is high-resistivity silicon with a permittivity of 11.9 and an electric conductivity of 0.00025 S·m^−1^.

### Experiment setup and fabrication methods

The prototypes are constructed using standard printed circuit board (PCB) technology on substrates with relative a permittivity of 6.15 and a loss tangent of 0.002. The rectangular waveguide is replaced by SIW, which is equivalent to a rectangular waveguide but avoids bulky volumes. For the circuit with a positive inductor, the metallic post is realized by perforating a via-hole between top metal and bottom metal, and behaves as a positive inductor. The metallic via-hole representing a positive inductive load is also applied in other prototypes. For the circuit with positive and negative inductors, besides the metallic via-hole as positive inductor, two rectangular apertures beside the metallic via-hole are cut off from the host substrate. Inside the cutoff rectangular apertures, two dielectric blocks composed of ceramic powder with a relative permittivity of 26.8 and a loss tangent of 0.002 are assembled to realize the negative inductor. For the circuit with a positive capacitor and inductor, besides the metallic via-hole as positive inductor, two layers of diaphragms are introduced to realize positive capacitors. For the convenience of the PCB technology, the equivalent diaphragms are achieved with metallic blind-hole fences, which are equivalent to the diaphragms. Two microstrip lines are adopted to transmit the input and output transmission in the waveguide, and the microstrip lines are connected to the Keysight N9917A vector network analyzer through the soldered SubMiniature Version A (SMA) connectors and coaxial cables. The S-parameters are derived from the analyzer.

### Supplementary information

Supplementary Information

## Data Availability

The simulation and experiment data that support the findings of this study are available from the corresponding author upon request.

## References

[1] Sadiku, M. N., & Alexander, C. K. *Fundamentals of Electric Circuits*, 5th edn (McGraw-Hill, 2013).

[2] Engheta N, Salandrino A, Alù A (2005). Circuit elements at optical frequencies: nanoinductors, nanocapacitors, and nanoresistors. Phys. Rev. Lett..

[3] Engheta N (2007). Circuits with light at nanoscales: optical nanocircuits inspired by metamaterials. Science.

[4] Zhang Q, Bai L, Bai Z, Hu P, Liu C (2015). Equivalent-nanocircuit-theory-based design to infrared broad band-stop filters. Opt. Express.

[5] Alù A, Young ME, Engheta N (2008). Design of nanofilters for optical nanocircuits. Phys. Rev. B.

[6] Sun Y, Edwards B, Alù A, Engheta N (2012). Experimental realization of optical lumped nanocircuits at infrared wavelengths. Nat. Mater..

[7] Caglayan H, Hong S-H, Edwards B, Kagan CR, Engheta N (2013). Near-infrared metatronic nanocircuits by design. Phys. Rev. Lett..

[8] Alù A, Engheta N (2008). Tuning the scattering response of optical nanoantennas with nanocircuit loads. Nat. Photonics.

[9] Huang J-S, Feichtner T, Biagioni P, Hecht B (2009). Impedance matching and emission properties of nanoantennas in an optical nanocircuit. Nano Lett..

[10] Liu N (2013). Individual nanoantennas loaded with three-dimensional optical nanocircuits. Nano Lett..

[11] Li J, Salandrino A, Engheta N (2007). Shaping light beams in the nanometer scale: a Yagi-Uda nanoantenna in the optical domain. Phys. Rev. B.

[12] Baba T, Ishihara T (2009). Analysis of transmission line metamaterials at optical wavelength. Phys. Status Solid..

[13] Song K, Mazumder P (2009). An equivalent circuit modeling of an equispaced metallic nanoparticles (MNPs) plasmon wire. IEEE Trans. Nanotechnol..

[14] Eleftheriades GV (2009). EM transmission-line metamaterials. Mater. Today.

[15] Monticone F, Estakhri NM, Alù A (2013). Full control of nanoscale optical transmission with a composite metascreen. Phys. Rev. Lett..

[16] Li Y, Liberal I, Engheta N (2016). Metatronic analogues of the Wheatstone bridge. J. Opt. Soc. Am. B.

[17] Silva A (2014). Performing mathematical operations with metamaterials. Science.

[18] Li H (2022). Performing calculus with Epsilon-near-zero metamaterials. Sci. Adv..

[19] Li Y, Liberal I, Giovampaola CD, Engheta N (2016). Waveguide metatronics: lumped circuitry based on structural dispersion. Sci. Adv..

[20] Collin, R. E. *Field Theory of Guided Waves* (McGraw-Hill, 1960).

[21] McGowan RW, Gallot G, Grischkowsky D (1999). Propagation of ultrawideband short pulses of terahertz radiation through submillimeter-diameter circular waveguides. Opt. Lett..

[22] Li, Y. & Engheta, N. Microwave analogues of multi-ordered metatronic filters with waveguide metamaterials. in *2016 URSI Asia-Pacific Radio Science Conference (URSI AP-RASC)*, 530–533 (2016).

[23] Li Y, Zhang Z (2018). Experimental verification of guided-wave lumped circuits using waveguide metamaterials. Phys. Rev. Appl..

[24] Sun W, Qin X, Wang S, Li Y (2022). General guided-wave impedance-matching networks with waveguide-metamaterial elements. Phys. Rev. Appl..

[25] Sun W, Qin X, Li H, Zhou Z, Li Y (2022). Impedance matching via ultrathin metatronic layer assisted by Smith Chart. Opt. Express.

[26] Chettiar UK, Engheta N (2015). Metatronic transistor amplifier. Phys. Rev. B.

[27] Promotion Group. IMT-2020 (5G) PG White paper, Beijing (2016).

[28] Hong W (2017). Multibeam antenna technologies for 5G wireless communications. IEEE Trans. Antennas Propag..

[29] Rotman W (1962). Plasma simulation by artificial dielectrics and parallel-plate media. IRE Trans. Antennas Propag..

[30] Qin X (2022). Waveguide effective plasmonics with structure dispersion. Nanophotonics.

[31] Qin X, Sun W, He Y, Zhou Z, Li Y (2022). Negative capacitors and inductors in optical plasmonic nanocircuits. Phys. Rev. B.

[32] Xu F, Zhang Y, Hong W, Wu K, Cui T (2003). Finite difference frequency-domain algorithm for modeling guided-wave properties of substrate integrated waveguide. IEEE Trans. Microw. Theory Tech..

[33] Yan L (2004). Simulation and experiment on SIW slot array antennas. IEEE Microw. Guide. Wave Lett..

[34] Scherer A, Painter O, Vuckovic J, Lončar M, Yoshie T (2002). Photonic crystals for confining, guiding and emitting light. IEEE Trans. NanoTechnol..

[35] Baba T (2007). Photonic crystals remember the light. Nat. Photonics.

[36] Johnson, S. G., Fan, S., Villeneuve, P. R., Joannopoulos, J. D. & Kolodziejski, L. A. Guided modes in photonic crystal slabs. *Phys. Rev. B* **60**, 5751 (1999).

[37] Noda S, Chutinan A, Imada M (2000). Trapping and emission of photons by a single defect in a photonic bandgap structure. Nature.

