# Supplementary material for: Negative capacitors and inductors enabling wideband waveguide metatronics
Source: Nat Commun. 2023 Nov 3;14:7041. doi: 10.1038/s41467-023-42808-z (PMC10624880; doi:10.1038/s41467-023-42808-z)
Supplement: Supplementary file 1 — Supplementary Information [file 41467_2023_42808_MOESM1_ESM.pdf]

Supplementary Materials for

Negative Capacitors and Inductors Enabling Wideband Waveguide Metatronics

Xu Qin[1], Pengyu Fu[1], Wendi Yan[1], Shuyu Wang[1], Qihao Lv[1] and Yue Li[1,2+]

[1]*Department of Electronic Engineering, Tsinghua University, Beijing 100084, China*

[2]*Beijing National Research Center for Information Science and Technology, Beijing 100084, China*

* lyee@tsinghua.edu.cn

Supplementary Note 1. The value of the lumped elements in waveguide metatronics.

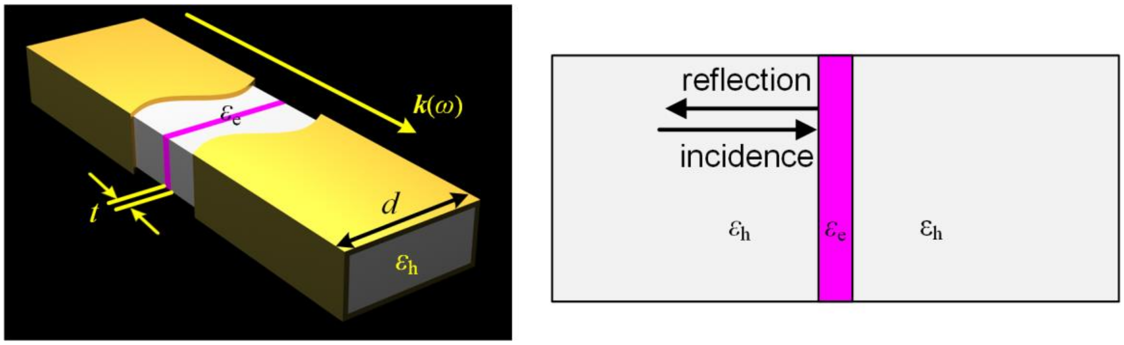

Supplementary Fig. 1. Sketches of the lumped elements in waveguide metatronics.

A dielectric slab with relative permittivity of ε_e is inserted in a waveguide host medium ε_h, which would lead to a discontinuity in the waveguide. The electromagnetic responses of the waveguide discontinuity could be written as follows[1]:

$$\frac{H}{E}=\sqrt{\frac{\varepsilon_0\varepsilon_h^{eff}}{\mu_0}}-i\omega(\varepsilon_e-\varepsilon_h)\varepsilon_0 t \quad (1)$$

where H and E is the total magnetic field and electronic field at the discontinuity, respectively. $\varepsilon_h^{eff}=\varepsilon_h-\frac{\pi^2c^2}{d^2\omega^2}$ represents the effective permittivity in the rectangle metallic waveguide according to the waveguide effective plasmonics[2]. Specifically, the characteristic admittance of a metallic waveguide[3] could be written as $Y_C=\frac{8d}{\pi^2h}\sqrt{\frac{\varepsilon_0\varepsilon_h^{eff}}{\mu_0}}$. Noted that $\sqrt{\frac{\varepsilon_0\varepsilon_h^{eff}}{\mu_0}}=\frac{H_i}{E_i}$, i.e. $Y_C=\frac{8d}{\pi^2h}\frac{H_i}{E_i}$ where H_i and E_i is the incident magnetic field and electronic field at the discontinuity, respectively. The characteristic admittance Y_C represents the non-reflection transmission in the metallic waveguide. According to the definition of the admittance in transmission lines, the admittance of a metallic waveguide, which represents the specific

electromagnetic state including total reflecting and transmitting waves, could be written as $Y=\frac{8d}{\pi^2 h}\frac{H}{E}$. Then from Supplementary Equation (1) we could derive the actual admittance in the waveguide as:

$$\begin{aligned} Y &= Y_{\mathrm{C}} - i\omega(\varepsilon_{\mathrm{e}}-\varepsilon_{\mathrm{h}})\varepsilon_0 t\frac{8d}{\pi^2 h} \\ &= Y_{\mathrm{C}} - i\omega(\varepsilon_{\mathrm{e}}-\varepsilon_{\mathrm{h}})\varepsilon_0 t\sqrt{\frac{\mu_0}{\varepsilon_0\varepsilon_{\mathrm{h}}^{\mathrm{eff}}}}Y_{\mathrm{C}} \end{aligned} \tag{2}$$

Then the normalized admittance is:

$$y = 1 - i\omega(\varepsilon_{\mathrm{e}}-\varepsilon_{\mathrm{h}})\varepsilon_0 t\sqrt{\frac{\mu_0}{(\varepsilon_{\mathrm{h}}-\frac{\pi^2c^2}{d^2\omega^2})\varepsilon_0}} \tag{3}$$

which means the electromagnetic response of the discontinuity could be modularized as a lumped element with normalized admittance:

$$y_{\mathrm{e}} = -\frac{i\omega(\varepsilon_{\mathrm{e}}-\varepsilon_{\mathrm{h}})\varepsilon_0 t}{\sqrt{\varepsilon_{\mathrm{h}}-\frac{\pi^2c^2}{d^2\omega^2}}}\sqrt{\frac{\mu_0}{\varepsilon_0}} \tag{4}$$

As we have analyzed in the main text, when $\Delta\varepsilon=\varepsilon_{\mathrm{e}}-\varepsilon_{\mathrm{h}}=Const.\times(1-\omega_{\mathrm{cut}}^2/\omega^2)$,then we have

$$\begin{aligned} y_{\mathrm{e}} &= -\frac{i\omega Const.\times(1-\omega_{\mathrm{cut}}^2/\omega^2)\varepsilon_0 t}{\sqrt{\varepsilon_{\mathrm{h}}-\frac{\pi^2c^2}{d^2\omega^2}}}\sqrt{\frac{\mu_0}{\varepsilon_0}} \\ &= -i\omega\varepsilon_0 tConst.\frac{(1-\omega_{\mathrm{cut}}^2/\omega^2)}{\sqrt{\varepsilon_{\mathrm{h}}-\frac{\pi^2c^2}{d^2\omega^2}}}\sqrt{\frac{\mu_0}{\varepsilon_0}} \\ &= -i\omega\varepsilon_0 tConst.\sqrt{\frac{\mu_0}{\varepsilon_0}}\sqrt{1-\frac{\omega_{\mathrm{cut}}^2}{\omega^2}} \\ &= -i\omega\tilde{C}\sqrt{1-\frac{\omega_{\mathrm{cut}}^2}{\omega^2}} \end{aligned} \tag{5}$$

where $\omega_{\text{cut}} = \dfrac{\pi c}{d\sqrt{\varepsilon_{\text{h}}}}$ is the cutoff angular frequency of the rectangle waveguide and $\tilde{C}$ is a constant for the value of the capacitor. Then the waveguide-modulated capacitor could be derived as $C = \sqrt{1 - \dfrac{\omega_{\text{cut}}^2}{\omega^2}}\tilde{C}$. As the sign of $\sqrt{1 - \dfrac{\omega_{\text{cut}}^2}{\omega^2}}$ is always positive (for a waveguide, the operating frequency is always higher than the cutoff frequency), the sign of the capacitor is dependent on the sign of $\tilde{C}$ and finally dependent on the sign of the $Const.$ in $\Delta\varepsilon = \varepsilon_{\text{e}} - \varepsilon_{\text{h}} = Const. \times (1 - \omega_{\text{cut}}^2 / \omega^2)$, which is exhibited in the main text. Similarly, when $\Delta\varepsilon = \varepsilon_{\text{e}} - \varepsilon_{\text{h}}$ is a constant, the normalized admittance could be fitted as a waveguide-modulated inductor, and the normalized admittance could have an approximate value as $y_{\text{e}} = -\dfrac{1}{i\sqrt{\omega^2 - \omega_{\text{cut}}^2}\tilde{L}}$, where $\tilde{L}$ is a constant for inductor value. Then the value of the lumped inductor could be derived as $L = \sqrt{1 - \dfrac{\omega_{\text{cut}}^2}{\omega^2}}\tilde{L}$. Similarly, the sign of the inductor is dependent on the sign of $\tilde{L}$. On the other hand, as a counterpart of the parallel lumped element, the series lumped elements could be derived directly from the dual theorem by creating permeability difference in the waveguide, and thus the comprehensive element map could be completed.

Supplementary Note 2. Detailed structure of the numerical analysis and experiment.

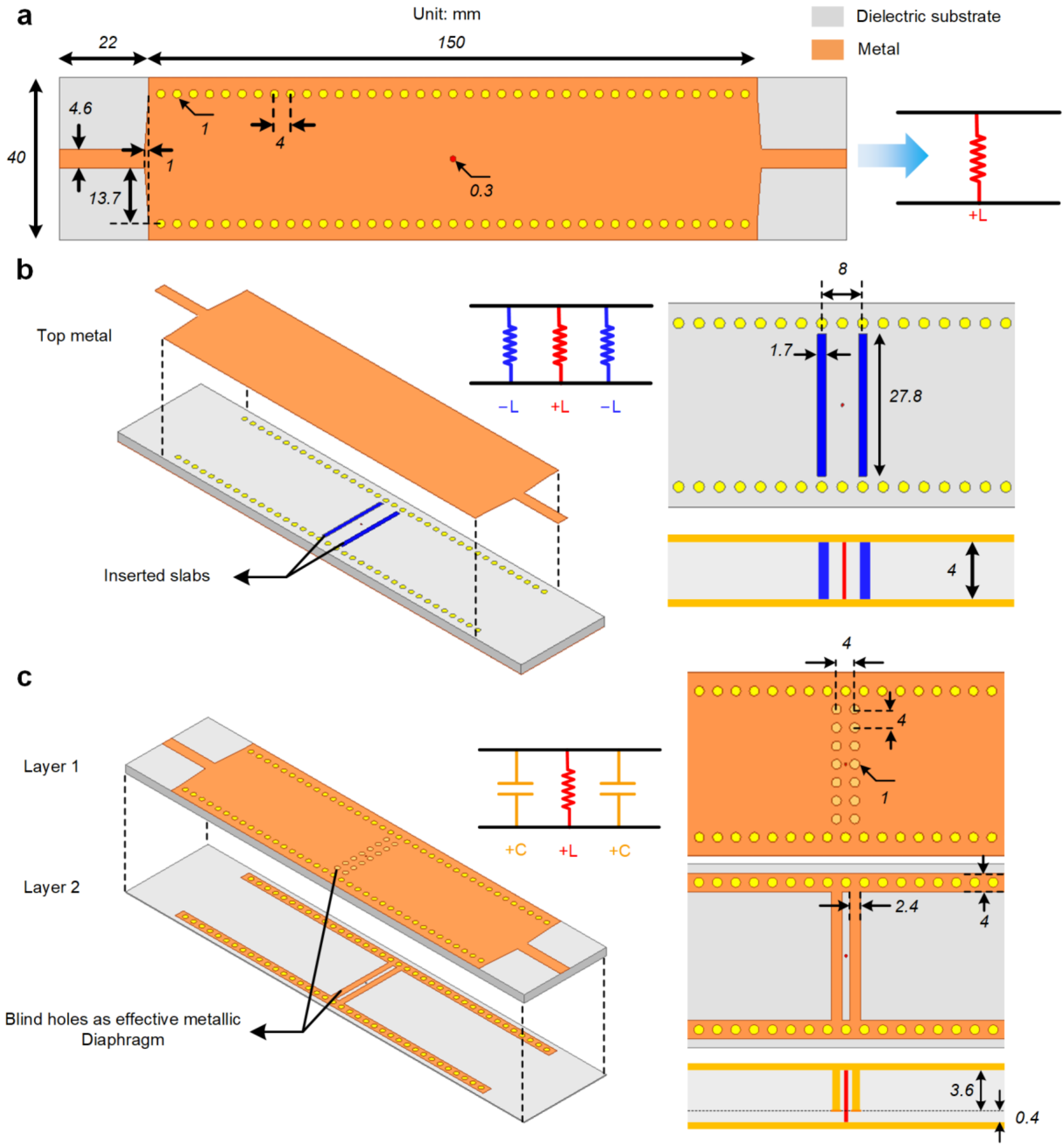

Supplementary Fig. 2. Detailed structure and setup of the numerical analysis and experiment.

Supplementary Fig. 2 exhibits the detailed structure and dimensions of the numerical analysis and experiment in the main text. Supplementary Fig. 2 (a) exhibits the waveguide platform for the waveguide metatronics and the metallic post behaving as a positive inductor. The permittivity of

the dielectric substrate is 6.15 with $tan\delta$=0.0019, and the waveguide is realized by the substrate integrated waveguide (SIW) with via fences equivalent to perfect electric conductor boundary. The cutoff frequency of the SIW is f_0=2 GHz. The SIW is connected to the network analyzer through 50-Ohm microstrip lines. At the center of the SIW, there is a metallic post that behaves as a positive inductor. The detailed dimensions of the SIW and the metallic post are labeled in Supplementary Fig. 2 (a). In Supplementary Fig. 2 (b), two inserted slabs with different permittivity from the host substrate cloaked the metallic post. The dimensions and position of the cloaking slabs are labeled in Supplementary Fig. 2 (b). The cloaking slabs are realized by ceramic powders with permittivity of $\varepsilon_{\text{slab}} = 26.8$. In Supplementary Fig. 2 (c), the metallic diaphragms behaving as positive capacitors are replaced by blind via fences which are equivalent to the metallic diaphragms[4]. The dimensions and positions of the via fences are labeled in Supplementary Fig. 2 (c). It is noted that in Figs. 2 (g-i) in the main text the lengths of the SIW are doubled to 300 mm to present clearer transmission performances and sharper comparisons.

Supplementary Note 3. Comparisons between the results and theoretical circuit models.

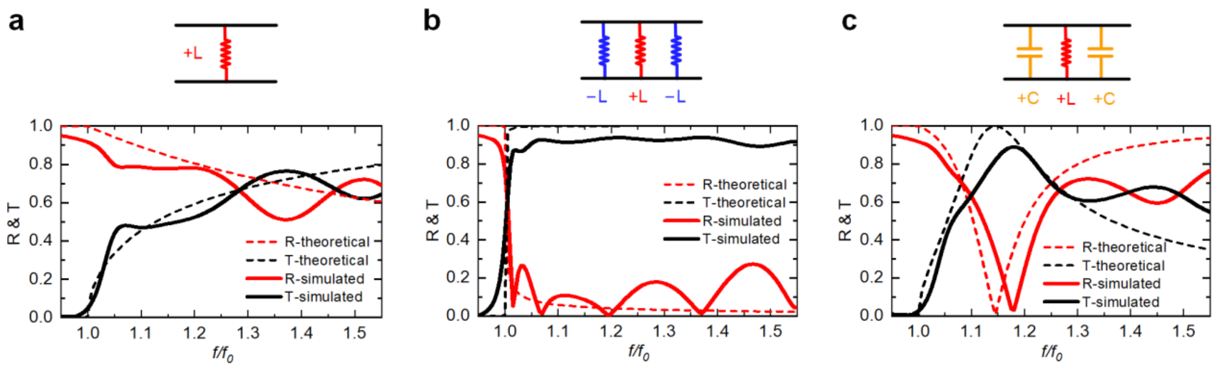

Supplementary Fig. 3. Comparisons of the reflection and transmission coefficients between the simulated results and theoretical circuit models.

Supplementary Fig. 3 exhibits the simulated results and the corresponding theoretical circuit model of the three kinds of circuits. In the theoretical circuit model, the dispersive values of the lumped circuit element are derived from curve fitting for each single lumped circuit element, and the values could be written as $L_+ = \sqrt{1-\frac{\omega_{\mathrm{cut}}^2}{\omega^2}}\tilde{L}_+$, $L_- = \sqrt{1-\frac{\omega_{\mathrm{cut}}^2}{\omega^2}}\tilde{L}_-$ and $C_+ = \sqrt{1-\frac{\omega_{\mathrm{cut}}^2}{\omega^2}}\tilde{C}_+$, respectively, where the cutoff frequency of the waveguide is $\omega_{\mathrm{cut}} = 2\pi\times 2\times 10^9\, rad\cdot s^{-1}$, and constants in the expression of each lumped circuit elements are $\tilde{L}_+ = 4.424\times 10^{-11} H\cdot\Omega^{-1}$ for the positive inductor in Supplementary Fig. 3 (a), $\tilde{L}_- = -2.147\times 10^{-11} H\cdot\Omega^{-1}$ for the two negative inductors in Supplementary Fig. 3 (b), $\tilde{C}_+ = 2.311\times 10^{-10} F\cdot\Omega$ for the two positive capacitors in Supplementary Fig. 3 (c). As shown in Supplementary Fig. 3, the reflection and transmission coefficients of the theoretical circuits model are consistent with the simulated results. The difference between the theoretical and simulated results probably comes from the unideal microstrip-waveguide transition and the material losses.

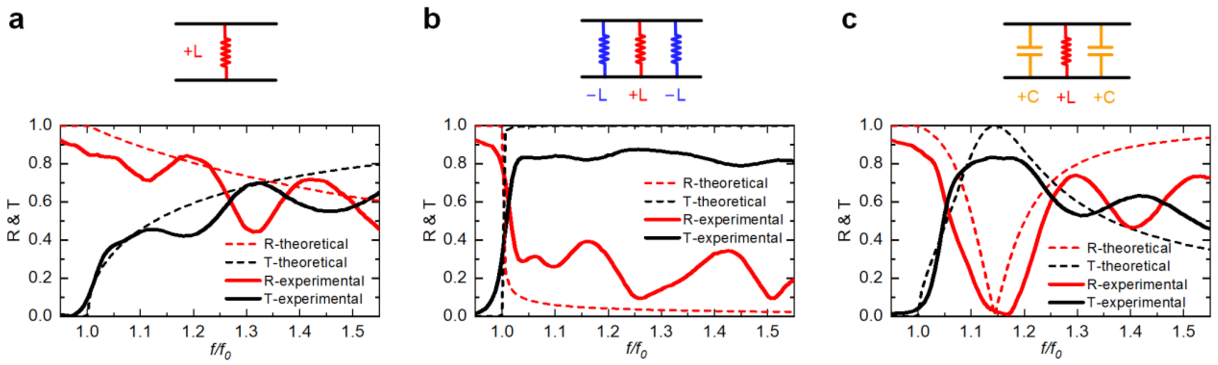

Supplementary Fig. 4. Comparison of the reflection and transmission coefficients between the experimental results and theoretical circuit models.

Supplementary Fig. 4 exhibits the experimental results and the corresponding theoretical circuit model of the three kinds of circuits. In the theoretical circuit model, the dispersive values of the lumped circuit element are derived from curve fitting for each single lumped circuit element, and the values could be written as $L_+ = \sqrt{1-\frac{\omega_{\text{cut}}^2}{\omega^2}}\tilde{L}_+$, $L_- = \sqrt{1-\frac{\omega_{\text{cut}}^2}{\omega^2}}\tilde{L}_-$ and $C_+ = \sqrt{1-\frac{\omega_{\text{cut}}^2}{\omega^2}}\tilde{C}_+$, respectively, where the cutoff frequency of the waveguide is $\omega_{\text{cut}} = 2\pi\times 2\times 10^9 rad\cdot s^{-1}$, and constants in the expression of each lumped circuit elements are $\tilde{L}_+ = 4.424\times 10^{-11} H\cdot\Omega^{-1}$ for the positive inductor in Supplementary Fig. 4 (a), $\tilde{L}_- = -2.147\times 10^{-11} H\cdot\Omega^{-1}$ for the two negative inductors in Supplementary Fig. 4 (b), $\tilde{C}_+ = 2.311\times 10^{-10} F\cdot\Omega^{-1}$ for the two positive capacitors in Supplementary Fig. 4 (c). As shown in Supplementary Fig. 4, the reflection and transmission coefficients of the theoretical circuits model are consistent with the experimental results. The difference between the theoretical and experimental results probably comes from the unideal microstrip-waveguide transition, the material losses, and the unideal experimental feeding.

Supplementary Note 4. Detailed structure of waveguide metatronics at terahertz.

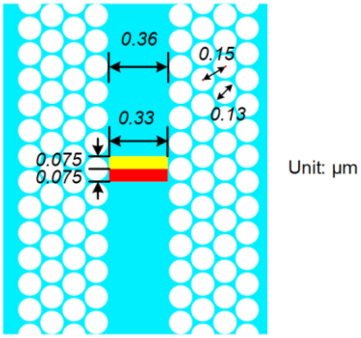

Supplementary Fig. 5. Detailed structure of waveguide metatronics and negative elements in silicon-based waveguide at terahertz.

Supplementary Fig. 5 exhibits the waveguide metatronics at terahertz. The thickness of the silicon substrate with permittivity of 11.9 is 0.3 μm, and the photonic crystal cladding is adopted as the boundary of the dielectric waveguide. The slabs are inserted in the silicon substrate behaving as the metatronics element pair. The dimensions of the silicon waveguide and the element pair are labeled in Supplementary Fig. 5. In the pair of positive inductor and negative inductor, the permittivities of the two slabs are 5 and 18.8, respectively. In the pair of positive inductor and positive capacitor, the positive inductor is realized by the slab with a permittivity of 5, while the permittivity of the slab behaving as a positive capacitor is Drude model $\varepsilon = \varepsilon_\infty - \omega_{\mathrm{p}}^2 / \omega^2$, where $\varepsilon_\infty = 60.9$ and $\omega_{\mathrm{p}} = 2\pi \times 1260 \times 10^{12} rad \cdot s^{-1}$.

Supplementary Note 5. Circuit implementations in silicon-based optical waveguide.

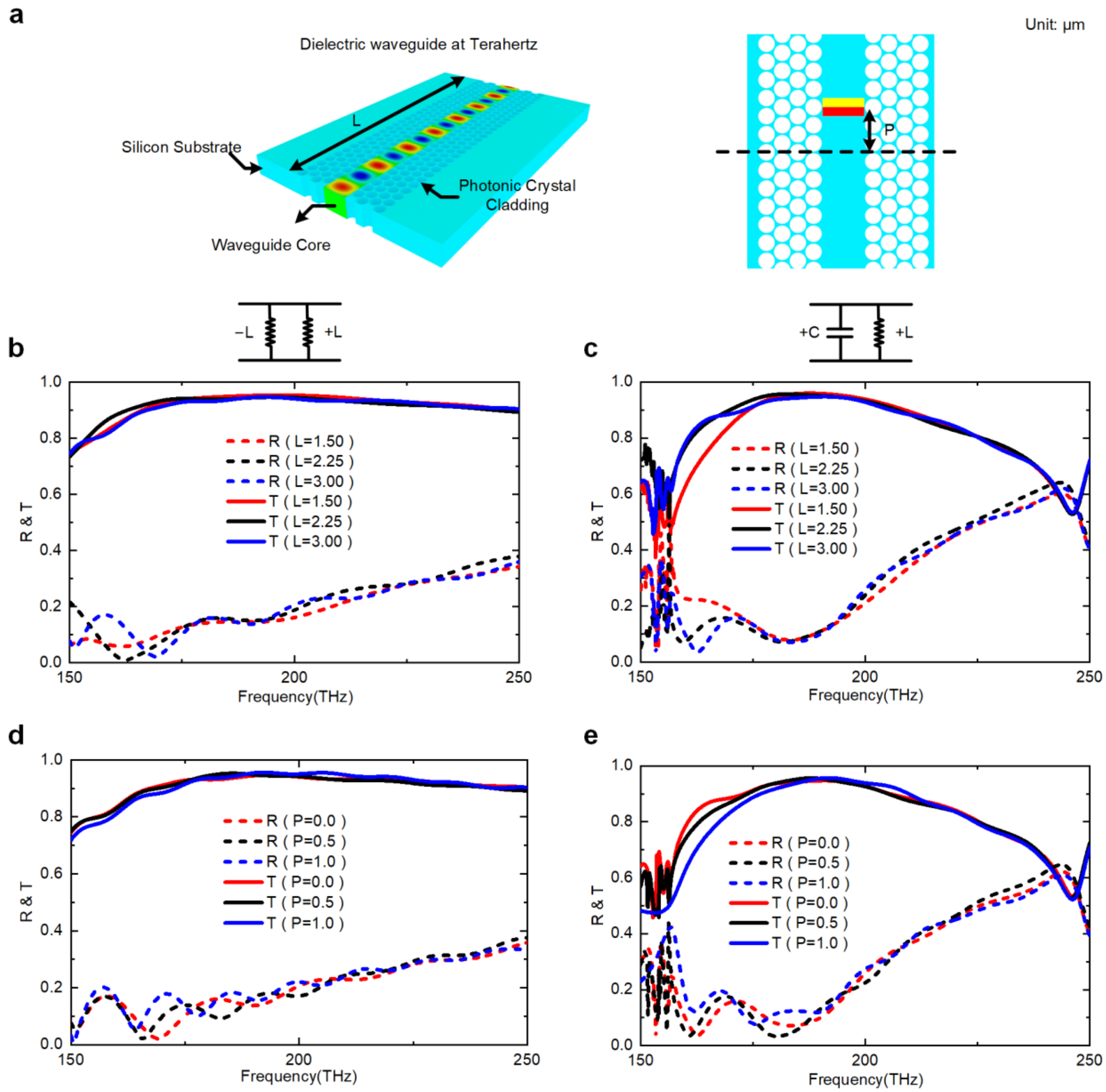

Supplementary Fig. 6. More examples of the circuit implementation in optical waveguides with varying lengths and asymmetric structures.

Supplementary Fig. 6 exhibits more examples of the circuit implementation in optical waveguide. Supplementary Fig. 6 (a) presents the length of the optical waveguide L and the offset distance P. In Supplementary Figs. 6 (b) and (c), there exhibits the transmission and reflection coefficients of

two kinds of lumped circuits with different lengths. In Supplementary Figs. 6 (d) and (e), there exhibits the transmission and reflection coefficients of the two lumped circuits with different offset distance. We can see the results remain consistent with different parameters, verifying the property of the circuit implementations in the optical waveguide.

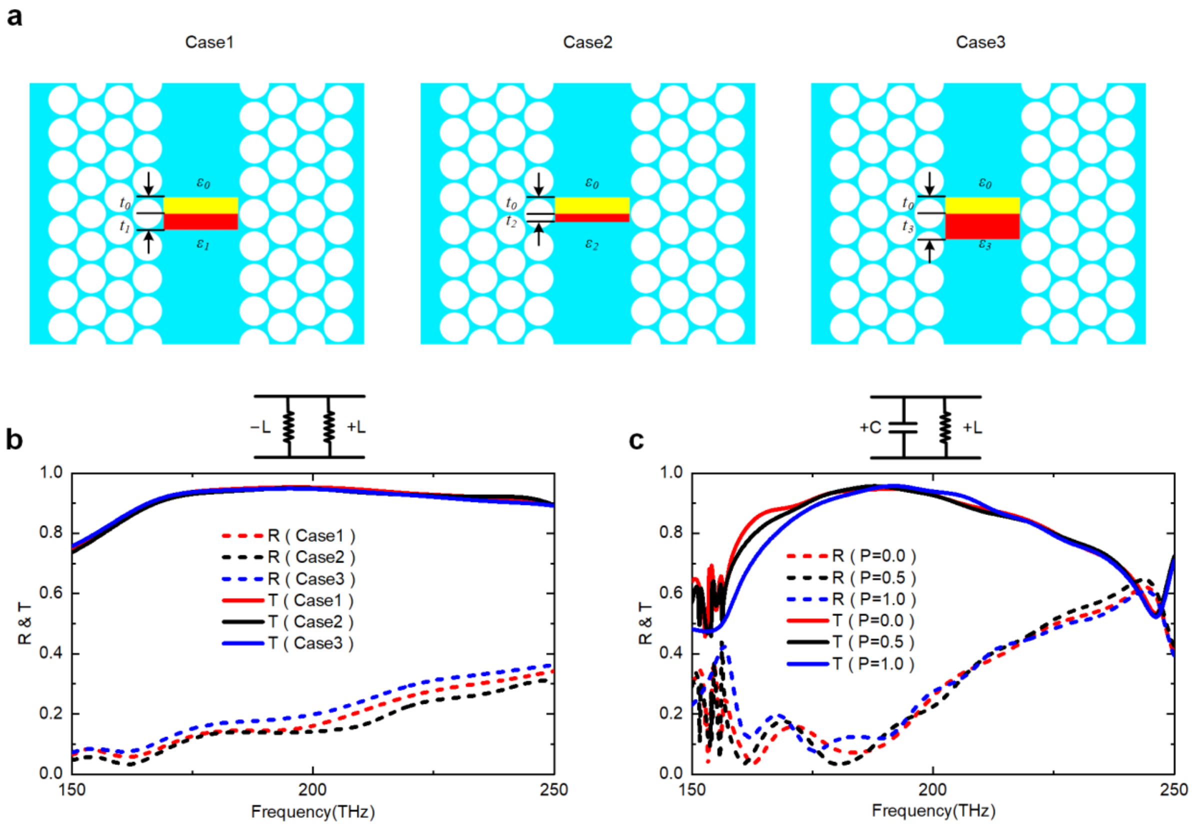

Supplementary Fig. 7. More examples of the circuit implementation in optical waveguides with different realizations of the lumped elements.

Supplementary Fig. 7 exhibits more examples of the circuit implementation in the optical waveguide. Supplementary Fig. 7 (a) presents three cases with different materials and sizes of lumped elements presenting the same value of the lumped positive inductors. Specifically, the parameters of the three cases are $t_1 = 0.075\mu m$, $t_2 = 0.05815\mu m$, $t_3 = 0.1056\mu m$, $\varepsilon_1 = 5$, $\varepsilon_2 = 3$

and $\varepsilon_3 = 7$ (so that $t\Delta\varepsilon$ could remain unchanged). The other structures and parameters of the two kinds of lumped circuits are the same as those in the main text. As shown in Supplementary Figs. 7 (b) and (c), in three cases of the two kinds of lumped circuits, the reflection and transmission coefficients remain consistent, verifying the value design of the lumped elements in the optical waveguide.

Reference

1. X. Qin, W. Sun, Y. He, Z. Zhou and Y. Li, 'Negative capacitors and inductors in optical plasmonic nanocircuits', *Physical Review B,* vol. 106, no. 16, pp. 165410, 2022.
2. X. Qin, W. Sun, Z. Zhou, P. Fu, H. Li and Y. Li, 'Waveguide Effective Plasmonics with Structure Dispersion', *Nanophotonics,* vol. 11, no. 9, pp. 1659-1676, 2022.
3. L.-S. Wu, B. Xia, J. Mao, and W.-Y. Yin, 'A half-mode substrate integrated waveguide Ring for two-way power division of balanced circuit', *IEEE Microw. Wireless Compon. Lett.* vol. 22, no. 7, pp. 333-335, 2012.
4. R. E. Collin, Field Theory of Guided Waves. (McGraw-Hill, New York, 1960).
